# How one treats lateral epicondylitis – a survey among Brazilian orthopedists

**DOI:** 10.1186/s12891-021-04445-9

**Published:** 2021-07-03

**Authors:** Rafael Fuchs Lazarini, Renato Arouca Zan, João Carlos Belloti, Ildeu Afonso de Almeida Filho, Luiz Fernando Sartori Centenaro, Fabio Teruo Matsunaga, Marcel Jun Sugawara Tamaoki

**Affiliations:** 1 Department of Orthopedics and Traumatology, Felicio Rocho Hospital, Belo Horizonte, MG Brazil; 2 Department of Orthopedics and Traumatology, Universidade Federal de São Paulo-Escola Paulista de Medicina (Unifesp-EPM), São Paulo, SP Brazil

**Keywords:** Lateral epicondylitis, Tennis elbow, Survey and questionnaires, Injections

## Abstract

**Background:**

Lateral epicondylitis (LE), also known as tennis elbow, is the most common painful elbow condition. It affects approximately 1–3% of adults. There are various possible treatments described in the literature, but evidence to support a gold standard management protocol is lacking. Therefore, the objective of this study was to evaluate how Brazilian orthopaedists diagnose and treat lateral epicondylitis and compare these results with the available evidence.

**Methods:**

This is an observational, analytical, cross-sectional study. A questionnaire was prepared to obtain information from the participants with eight specific questions (2 on diagnosis and 6 on treatment). These questions were answered voluntarily by participants at 3 major congresses of orthopaedists in Brazil in 2018. The results were analysed in accordance with the overall number of responses and were evaluated among groups according to subspecialty.

**Results:**

We obtained a total of 501 questionnaires. Of these, 33 were excluded. The mean age was 38.67 years. The majority of respondents (91%) were male. We obtained 26.7% from specialists in hand surgery (Hand group), 36.5% from subspecialists in shoulder and elbow (Shoulder and Elbow group), and 36.8% from generalists in orthopaedics or from other subspecialties (General Orthopaedists group). For diagnosis, 24.4% did not initially request any imaging method. The most requested exam was ultrasonography (54.9%). The most prominent indication for initial treatment was physical therapy. For refractory cases, 78.3% of the respondents preferred doing a local infiltration. The most commonly used substance for local infiltrations was corticosteroids (89.6%). With respect to the surgical treatment option, 75.8% of those who recommend it prefer open techniques, and 24.2% prefer arthroscopic treatment. Of the total respondents, 12.8% did not recommend surgical treatment for LE.

**Conclusion:**

Among Brazilian orthopaedists, the Cozen test is most frequently chosen, and ultrasound is the most commonly used imaging tool. Nonsurgically, oral nonsteroidal anti-inflammatory drugs (NSAIDs) plus physiotherapy is the most popular initial therapy, and corticosteroids are the most popular type of infiltration agent. Most surgeons recommended surgery after 6 months of nonsurgical treatment, and 75.8% preferred the open technique.

## Background

Lateral epicondylitis (LE), or tennis elbow, is widely studied because it is the most common painful condition of the elbow. It affects approximately 1–3% of adults [1] and has a higher incidence in patients whose activities require repetitive or excessive efforts [2]; therefore, it is an important cause of absenteeism [1], leading to a socioeconomic impact. Given this context, there is a large number of studies on the subject [3–13]; in a quick search of the PubMed database in 2021, using the term LE, 2571 studies were identified. However, there is uncertainty about the best way to manage this condition. Despite its prevalence and morbidity, there is still no single gold standard management guide for the disorder.

The diagnosis of LE is primarily clinical. Tenderness over the lateral epicondyle is a common finding [14]. The Cozen test reproduces ﻿pain over the epicondyle, with active resistance to extension of the wrist and pronation of the forearm [14]. The Mills test [15] starts with the elbow at 90° of flexion, and the examiner passively flexes the wrist and extends the elbow, while the Maudsley test [16] is performed by resisting extension of the third finger. All of these tests are positive in the presence of lateral epicondylar pain [14]. However, imaging tests can help when addressing referred pain. Conventional radiographs, ultrasound, and magnetic resonance imaging can be used to exclude osteoarticular disease, tendon injury, cartilage damage, synovial plica, and loose bodies, but no clinical guidelines are set as the gold standard.

Nonsurgical treatment includes patient education, behavioural modification, nonsteroidal anti-inflammatory drugs (NSAIDs), physical therapy, and orthoses. In addition, shock waves, laser therapy, ultrasound, and local infiltrations are options. Surgical treatment, such as open [17] or arthroscopic [18] treatment, can be used, especially for refractory cases. Many authors have suggested treatment guidelines [19], but no gold standard has been established.

Due to the myriad of options to address this condition and the conflicting results of various therapies [19–22], and given that there is no single flowchart based exclusively on scientific evidence, treatment in clinical practice is quite extensive and dependent on the choice and experience of the attending physician. Thus, it can be helpful to identify diagnosis and treatment patterns in a country, even if a gold standard is not available. Therefore, the objective of this study is to appraise how Brazilian orthopaedists diagnose and treat lateral epicondylitis.

## Methods

This is an observational, analytical, cross-sectional study. A questionnaire comprising eight specific questions, two on diagnosis and six on treatment, is summarized in Table 1. The questions were answered voluntarily by participants at the 38th Brazilian Congress on Hand Surgery (***MÃO***), at the 12th Brazilian Congress on Shoulder and Elbow Surgery (***CBCOC***), and at the 50th Brazilian Congress on Orthopaedics and Traumatology (***CBOT***) in 2018.

**Table 1:** The table summarizes the specific questions related to the diagnosis and treatment of lateral epicondylitis, with the number of responses and possible alternatives that could be checked

Question	Number of possible responses	Alternatives
**Which complementary exam(s) do you use for diagnosis?**	multiple	none, radiography, ultrasound, magnetic resonance, or other
**Which manoeuver(s) do you use in the physical exam?**	multiple	palpation, Cozen, Mill's, Maudsley, other
**What is(are) your option(s) for initial treatment?**	multiple	Physiotherapy, Orthosis, Rest, Local infiltration, Surgery, Oral anti-inflammatory, Intramuscular anti-inflammatory
**How long after initial treatment do you recommend a change in treatment?**	multiple	Up to 1 month, 3–6 months, 6–12 months, >12 months
**If symptoms persist, what is your treatment option?**	single	Physiotherapy, Orthosis, Rest, Local infiltration, Surgery, Oral anti-inflammatory, Intramuscular anti-inflammatory
**When performing local infiltration, which is your choice substance?**	single	do not perform, botulinum toxin, corticosteroids, autologous blood, sodium hyaluronate, prolotherapy, only anaesthetics
**How long after conservative treatment do you recommend surgery?**	single	do not recommend, 3 months, 6 months, 12 months
**What is your surgery option for lateral epicondylitis of the elbow?**	single	open, arthroscopic

We included participants from the mentioned congresses, who were members of the Brazilian Society of Orthopaedics and Traumatology (***SBOT***), who agreed to answer the questionnaire. Participants from other nationalities, nonmedical participants, duplicated questionnaires, those with more than 3 specific answers not filled out, or those without identification were excluded.

For analysis of the results, participants were divided into 3 groups based on training: hand specialists (Hand group), shoulder and elbow specialists (Shoulder and Elbow group), and general practitioners or orthopaedists with other subspecialties that may occasionally also treat a patient with lateral epicondylitis in office (General Orthopaedists group). A comparative analysis was carried out among the groups to assess the preference profile.

We tested the normality of the main outcome quantitative variables using the Kolmogorov-Smirnov (KS) test and verified a normal distribution. The overall quantitative answers were evaluated by the two proportions test, and qualitative answers were evaluated by ANOVA. To compare the answers among groups, ANOVA was employed using variance of quantitative parameters, and the chi-square test was used to assess possible statistical associations among qualitative responses. Significance was verified when the *p*-value of the results was <0.001. The confidence level of the results was established with *p* < 0.05, and the confidence interval was 95%.

## Results

We obtained a total of 501 questionnaires. Of these, 10 were excluded for presenting three or more incomplete answers; 16 were not included because they were from nonmedical respondents; four were foreigners; and one had been answered by the same person at different congresses. Of the 468 evaluated, 38.5% were obtained at *CBOT* 2018, 35.9% at *CBCOC* 2018, and 25.6% at *MÃO* 2018. The majority of participants (91%) were male.

The analysis of the evaluated questionnaires gave a 95% confidence interval and a margin of error of 4%, based on the 17,701 orthopaedists registered in the Brazilian Society of Orthopaedics and Traumatology (SBOT) in March 2018. Based on the 896 members of the Brazilian Society of Surgery of Shoulder and Elbow (SBCOC), the results for the 171 (36.5%) specialists in shoulder and elbow surgery gave a margin of error of 6.75%. Similarly, for the 734 members of the Brazilian Society of Hand Surgery (SBCM), the results for the 125 (26.7%) specialists in hand surgery gave a margin of error of 7.99%. The remaining 172 (36.8%) respondents, with a different fellowship training, were allocated to the general orthopaedist group.

### Diagnosis

The median number of manoeuvres performed in clinical diagnosis was 3, the most common being the Cozen test [14] (80.1%), local palpation [14] (74.6%), and Mill's test [15] (60.2%). The Maudsley test [16] had the lowest indication with 31.3% of participants. There was a statistically significant difference among the groups of specialties for the mean number of manoeuvres (*p*-value <0.001). Thus, Tukey’s multiple comparison (post hoc) was used to compare the groups in pairs. Differences were observed between the General Orthopaedists group, with the lowest mean for number of manoeuvres, at 1.99, compared with the Hand group, with a mean of 2.75 (*p*-value <0.001), and compared with the Shoulder and Elbow group mean of 2.92 (*p*-value <0.001).

For imaging diagnosis, 24.4% did not initially request any imaging method. The most commonly requested exam was ultrasound, indicated in 54.9% of the responses. The associations between the number of exams and the types of exams requested are shown in Table 2. Nineteen percent of general orthopaedists request an exam, while 31.2% of the hand group and 31% of the shoulder and elbow group request an exam.

**Table 2:** Relationship among the number of exams ordered, percentage in the sample, and results stratified by the combination of exams and their representativeness in the number of exam groups

Number of exams ordered	Group percentage (number of exams)	Result stratified by exam types	Percentage of combinations in relation to the total
**None**	**24.4%**	None	24.4%
**1 exam**	**40.4%**	XR	5.1%
		US	25.9%
		MRI	9.4%
**2 exams**	**23.5%**	XR + US	7.3%
		XR + MRI	6.2%
		US + MRI	10.0%
**3 exams**	**11.7%**	XR + US + MRI	11.7%
**TOTAL:**	100.0%		100.0%

*XR* radiographs, *US* ultrasound, *MRI* magnetic resonance imaging

### Treatment

**Chart 1:**
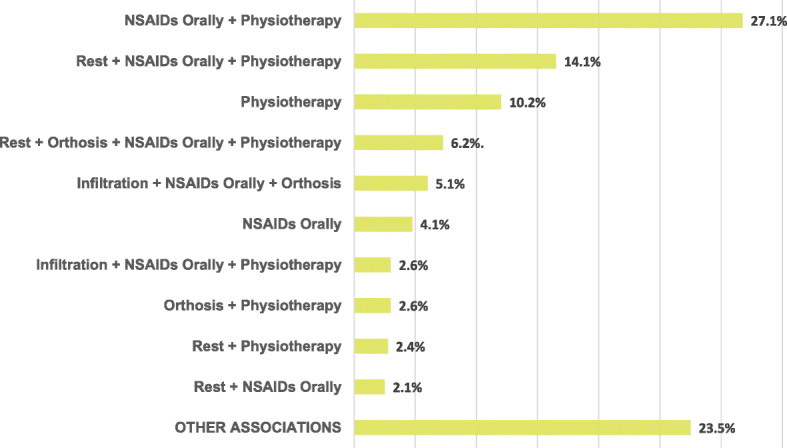
Most frequent associations in the initial treatment of lateral epicondylitis. In the caption, the percentages are the sample related to total answers. NSAIDs Orally: oral nonsteroidal anti-inflammatory drugs. OTHER ASSOCIATIONS refers to the sum of other associations answered with a frequency of less than 2%

Regarding the choice of initial treatment, the two most common interventions were physical therapy (86.7%) and the use of oral anti-inflammatory drugs (NSAIDs) (74.1%). Another relevant data point is that 11.3% of participants performed local infiltration as first-line therapy. The General Orthopaedist group indicated the use of oral NSAIDs more frequently than the Hand or Shoulder and Elbow specialists (*p* < 0.001). Furthermore, the Hand group prescribed more orthoses than the other groups (*p* < 0.001). No participant recommended surgery initially. The most frequent associations of overall responses are shown in Chart 1.

Regarding the period until treatment change, for persistent cases, the General Orthopaedist group change was 21.6% in the first month and 51.5% in 1–3 months. The Shoulder and Elbow group tended to wait for a longer period of time, with the most frequent answer (44.6%) being 3 and 6 months. With respect to the new treatment, 78.3% of the overall respondents preferred to perform local infiltration, and 14.4% preferred surgery.

The substances most frequently used for local infiltration, both initially and with the persistence of symptoms, were corticosteroids (89.6%). The subspecialists in the Hand and General Orthopaedists groups used corticosteroids more than 90% of the time, while 83.1% of subspecialists in the Shoulder and Elbow group did so, and the difference was not significant. Sodium hyaluronate (4.8%), platelet-rich plasma (0.7%), local anaesthetics only (0.7%), and botulinum toxin (0.2%) were also mentioned.

Respondents were then asked how long nonsurgical treatment was maintained. Most of them said they recommend surgical treatment in cases of refractory response to conservative treatment after 6 months (55.1%). Regarding the other possible answers, 10.8% recommended it after 3 months and 34.1% after 12 months.

With respect to the surgical treatment option, 12.8% did not recommend it. Regarding those who did recommend surgery, 75.8% preferred open techniques, and 24.2% preferred arthroscopic treatment. The Hand group indicated open operative treatment more frequently than the other groups (*p* < 0.001). The comparative analysis among groups regarding these questions is shown in Table 3.

**Table 3:** Distribution of specific questions by subspecialty group (n: number of responses, %: percentage)

GROUPS:		Hand		Shoulder and Elbow		General Orthopaedists		Total	
QUESTION:	ANSWER:	N	%	N	%	N	%	N	%
How long after the initial treatment do you recommend a change in treatment?	Up to 1 month	10	8.1%	9	5.4%	37	21.6%	56	12.1%
	1–3 months	53	42.7%	57	33.9%	88	51.5%	198	42.8%
	3–6 months	41	33.1%	75	44.6%	37	21.6%	153	33.0%
	6–12 months	14	11.3%	21	12.5%	8	4.7%	43	9.3%
	>12 months	6	4.8%	6	3.6%	1	0.6%	13	2.8%
If symptoms persist, what is your treatment option?	Surgery	22	17.6%	27	16.1%	18	10.5%	67	14.4%
	Local Infiltration	94	75.2%	130	77.4%	140	81.4%	364	78.3%
	Other	9	7.2%	11	6.5%	14	8.1%	34	7.3%
When infiltrating, which is your substance of choice?	Corticosteroids	109	91.6%	133	83.1%	153	94.4%	395	89.6%
	Sodium Hyaluronate	2	1.7%	15	9.4%	4	2.5%	21	4.8%
	Platelet-Rich Plasma	0	0.0%	1	0.6%	2	1.2%	3	0.7%
	Anaesthetics and perforations only	7	5.9%	11	6.9%	3	1.9%	21	4.8%
	Botulinum Toxin	1	0.8%	0	0.0%	0	0.0%	1	0.2%
How long after conservative treatment do you recommend surgery?	3 months	8	7.6%	10	6.6%	26	17.2%	44	10.8%
	6 months	59	56.2%	84	55.3%	82	54.3%	225	55.1%
	12 months	38	36.2%	58	38.2%	43	28.5%	139	34.1%
What is your surgery option for lateral epicondylitis of the elbow?	Open	104	91.2%	120	73.2%	105	67.3%	329	75.8%
	Arthroscopic	10	8.8%	44	26.8%	51	32.7%	105	24.2%

## Discussion

This is the first study to assess how Brazilian orthopaedists diagnose and treat lateral epicondylitis. Similar surveys have been conducted in other populations of professionals [23–25] to determine how lateral epicondylitis is managed. We can compare our results to those of other populations, although differences may be related to doctors preferences or health care systems of countries.

The diagnosis of lateral epicondylitis (LE) is clinical. The Cozen test, Mills test and palpation are widely used, although there is a lack of studies examining accuracy of these tests [26]. Ultrasound is the preferred imaging method for diagnosis, indicated by 54.9%, in contrast to 4% usage of American Fellowship-trained upper extremity surgeons before an indication for surgery [23]. Physiotherapy and oral NSAIDs are the main recommendations for first-line therapy, and Amar et al. [25] also found that these modalities are the most commonly indicated. Orthoses are more frequently indicated for hand surgeons, which we verified, and are a frequent indication for american surgeons (68% counterforce brace, 48% wrist brace) [23].

Infiltration is very frequently used as a second-line therapy by 78.3%, especially with corticosteroids (CS). Another survey showed the use of CS in infiltrations by approximately 71% of professionals in the United States [23]. In the United Kingdom, 21% use it routinely for most patients, and only 40% never use it [24]. In a survey of surgeons worldwide, 38% recommended infiltration with CS as a first-line therapy [25]. The results of our work corroborated that despite the evidence, the practice is still widely used in our country, even more frequently than in other populations. Although CS was the main substance chosen by our participants, there is evidence against the practice [27–29].

Surgery was not recommended by 12.8% of respondents and was performed using open techniques by the majority of respondents (75.8%). Additionally, most of the respondents wait at least 6 months before surgery. In a study carried out with upper limb specialists in the USA, 5% of participants did not recommend surgery, and of those who did, 75% preferred the open technique [23]. Among surgeons in the United Kingdom, 11% never recommend surgery, with the majority waiting at least 12 months for this indication [24]. In a survey of surgeons worldwide, only 10% of them recommended surgical treatment [25]; however, the treatment time was not evaluated, nor were the first- and second-line treatments differentiated in this study.

As a limitation of this study, we evaluated only participants of congresses and members of the *SBOT*. However, this study provided a representative sample of how Brazilian orthopaedists treat this pathology. We also did not include other professionals who can treat LE in the study, such as family doctors, occupational doctors, and rheumatologists. The questions were presented in a generic way, as it is very difficult to administer a questionnaire that involves all possible treatment variables, such as patient requirements and symptom intensity. In addition, when comparing our results with findings in other populations [23–25], we cannot explain whether these differences were due to personal preferences or could be influenced by differences in the health system or insurance system in each country.

As a strength of this work, we publish data that can relate the diagnosis and treatment options in our country with the evidence in the literature. This type of evaluation is important to warn about possible discrepancies. In addition, it can serve as a reference for the development of comparative studies, especially when there is no definitive evidence about which interventions are best, as in the case of LE treatment. Another positive point is the possibility of comparing these practices with those of other populations, although there are few studies on the subject, such as those already mentioned [23–25].

## Conclusion

Among Brazilian orthopaedists, the Cozen test is most frequently chosen, and ultrasound is the most commonly used imaging tool. Nonsurgically, oral NSAIDs plus physiotherapy is the most popular initial therapy, and corticosteroids are the most popular type of infiltration agent. Most surgeons recommend surgery after 6 months of nonsurgical treatment, and 75.8% prefer the open technique.

## Data Availability

The datasets that will be used and/or analyzed during the current study will be available from the corresponding author on reasonable request.
